# Laboratory Performance of Five Selected Soil Moisture Sensors Applying Factory and Own Calibration Equations for Two Soil Media of Different Bulk Density and Salinity Levels

**DOI:** 10.3390/s16111912

**Published:** 2016-11-15

**Authors:** Svatopluk Matula, Kamila Báťková, Wossenu Lemma Legese

**Affiliations:** 1 Department of Water Resources, Faculty of Agrobiology, Food and Natural Resources, Czech University of Life Sciences, Kamýcká 129, 165 21 Praha 6—Suchdol, Czech Republic; batkova@af.czu.cz (K.B.); Wos_Lemma@yahoo.com (W.L.L.); 2 Department of Water Supply and Sanitary, School of Environment and Energy Resource Engineering, Addis Ababa Science and Technology University, P.O. Box 16417, Addis Ababa, Ethiopia

**Keywords:** water content sensors, impedance, Frequency Domain Reflectometry (FDR), performance, factory calibration, own calibration, comparison, bulk density, salinity, sand, soil

## Abstract

Non-destructive soil water content determination is a fundamental component for many agricultural and environmental applications. The accuracy and costs of the sensors define the measurement scheme and the ability to fit the natural heterogeneous conditions. The aim of this study was to evaluate five commercially available and relatively cheap sensors usually grouped with impedance and FDR sensors. ThetaProbe ML2x (impedance) and ECH_2_O EC-10, ECH_2_O EC-20, ECH_2_O EC-5, and ECH_2_O TE (all FDR) were tested on silica sand and loess of defined characteristics under controlled laboratory conditions. The calibrations were carried out in nine consecutive soil water contents from dry to saturated conditions (pure water and saline water). The gravimetric method was used as a reference method for the statistical evaluation (ANOVA with significance level 0.05). Generally, the results showed that our own calibrations led to more accurate soil moisture estimates. Variance component analysis arranged the factors contributing to the total variation as follows: calibration (contributed 42%), sensor type (contributed 29%), material (contributed 18%), and dry bulk density (contributed 11%). All the tested sensors performed very well within the whole range of water content, especially the sensors ECH_2_O EC-5 and ECH_2_O TE, which also performed surprisingly well in saline conditions.

## 1. Introduction

Non-destructive soil water content determination is a fundamental component of agricultural applications such as precision agriculture and irrigation scheduling [1,2]. Several techniques exist for automated continuous point-scale soil water content measurements. For field application and sensor network realization, the costs of a single sensor need to be minimized and the lifetime needs to be maximized [3]. This study is focused on techniques based on dielectric characterization of the soil. Soil dielectric permittivity determination is applied in a variety of soil water sensing probes like Time Domain Reflectometry (TDR) [4,5,6,7] and Frequency Domain Reflectometry (FDR) [8,9,10,11,12,13]. Two publications related to sensors and their calibration and measurement accuracy for different soil conditions and influence of organic matter content were presented in 2016 [14,15]. Of these two methods, FDR is the more recent method and offers relatively accurate measurements for reasonable costs. The FDR and capacitance methods represent alternatives to TDR, which performs precisely but at a high price. They offer the possibility of continuous measurement and good applicability to various soils [16,17,18]. The disadvantage of the FDR sensors, which is often mentioned, is their high susceptibility to soil environmental effects [19,20]. The sensitivity of soil water content determination is mainly affected by parameters like the frequency of measurement, temperature, soil texture, soil bulk density, organic matter content, and electric conductivity. Chen and Or [19] concluded that the optimum frequency for measurement is 100 MHz. This frequency minimizes the effect of the so-called Maxwell–Wagner polarization.

Laboratory and field testing for different soil types is usually carried out by the producers of the sensors during the sensor development process. The producers usually provide customers with the results of tests for certain generalized basic soil types to cover soils from the whole world. This is why a lot of attention is paid to site- and purpose-specific calibrations e.g., [8,13,15,21,22,23,24,25,26,27]. The aim of such calibration is to obtain soil water content data with desirable accuracy. In order to obtain the highest possible accuracy, the abovementioned parameters need to be considered. It can be useful to identify a contribution of each parameter being considered. This knowledge can be helpful in a standard calibration methodology development. Although this aim has been discussed several times in specialized conferences [25,26], no standard calibration methodology has been presented yet. The aim of this study was not only to calibrate ThetaProbe ML2x, ECH_2_O EC-10, ECH_2_O EC-20, ECH_2_O EC-5, and ECH_2_O TE sensors, but also to contribute to the knowledge about the different factors affecting measurement accuracy.

Sensors based on a capacitance technique consist of a capacitor or condenser as a passive electronic component, consisting of a pair of conductors separated by a dielectric medium, while capacitance can be defined as the ability of two conductors to store charge when a voltage is applied across them [9]. Decagon Devices, Inc. (Pullman, WA, USA) developed various sensors based on a capacitance technique [28]; e.g., sensors from the ECH_2_O family EC-10, EC-20, EC-5, and ECH_2_O TE. The soil water content sensors are available in many varieties differing in length, size, and shape and with additional features such as temperature and electrical conductivity measurements. Factory calibrations show high variation in accuracy for different textural classes of soil and electrical conductivity [21,29,30]. Bogena et al. [3] published an evaluation of two low-cost soil water content sensors, ECH_2_O EC-5 and ECH_2_O EC-20 (Decagon Devices Inc., Pullman, WA, USA) in comparison with a TDR probe under laboratory and field conditions. Capacitor sensors usually use measuring frequencies between 5 and 100 MHz. In this frequency range, the imaginary part of permittivity is significantly high, which is mainly due to the effect of high ionic conductivity and dielectric absorption [31,32]. Hence the sensors are highly affected by the electrical conductivity or salinity of the soil. Furthermore, the effect of temperature and the air gap between the probe sensing body and soil is significant [13].

Sensors based on an impedance technique determine the impedance of a coaxial transmission line, which depends on its physical dimensions and the dielectric permittivity of the soil porous medium. Gaskin and Miller [10,12] conducted initial research on the potential use of the impedance technique to determine soil water content. The Macaulay Land Use Research Institute in Aberdeen, Scotland has developed the probe and Delta-T Devices Ltd. (Cambridge, UK) produces it as a ThetaProbe ML2x for volumetric water content measurement. The ThetaProbe generates a 100 MHz sinusoidal signal and outputs the measured impedance of the sampling medium as an analogue DC voltage between 0 and 1 V. The 100 MHz signal frequency was chosen to minimize the effect of ionic conductivity [12]. The soil sampling volume consists of a cylindrical four-signal rod array approximately 4 cm in diameter and 6 cm long, surrounding a center signal rod [12,33].

The performance of the soil water sensor can be evaluated in terms of resolution, sensitivity, repeatability, precision, and accuracy. Resolution is commonly assumed to be the smallest change in the measured quantity that can be detected in the output reading [34]. Resolution can be expressed either as a proportion of the reading or in absolute terms [35]. Sensitivity is generally defined as the minimum input of physical parameter that will create a detectable output change [35] or the ratio of the change in sensor output to a change of the quantity to be measured [34]. Repeatability (sometimes called reproducibility) can be described as the degree to which an experiment can be accurately replicated by someone else. It can be used to describe the ability of a sensor to provide the same result under the same circumstances [36]. Precision is usually defined as the ability of a measurement to be consistently repeated. An ideal sensor would measure exactly the same value for a number of repeated measurements. The real sensors output a range of values spread close to the actual correct value [35]. The accuracy of the sensor can be defined as the maximum difference that will exist between the measured value and the true (real, actual) value determined by a standard reference procedure [35]. In other words, it is the ability of a measurement to match the actual value of the measured quantity.

In this study, the performance of five soil water content sensors, (i) impedance soil water probe ThetaProbe ML2x (Delta-T Devices, Ltd., Cambridge, UK); and FDR (ii) ECH_2_O EC-10; (iii) ECH_2_O EC-20; (iv) ECH_2_O EC-5; and (v) ECH_2_O TE (Decagon Devices Inc., Pullman, WA, USA), was evaluated in terms of the accuracy in comparison with a standard gravimetric method to determine the water content in soils. Although a new version of ThetaProbe (ML3) and new sensors made by Decagon are being produced, the tested sensors are still widely used in praxis and research. Factory-set and our own calibration equations were applied in order to evaluate the ability of the sensor to determine the water content of artificially prepared soil profiles of three different dry bulk densities. The sensors were tested in two porous materials (silica sand, loess) and the effect of electrical conductivity was also considered. The use of silica sand ensured repeatable creations of profiles of different water contents/electrical conductivity, so the accuracy of the measurement was not affected by other factors such as the non-homogeneity of the profiles. The effect of temperature was minimized by performing the experiments under controlled laboratory conditions at a constant temperature of 20 °C.

## 2. Materials and Methods

### 2.1. Methods to Determine Soil Water Contents

#### 2.1.1. Referential Method

The water content determination by standard gravimetric method was used in this study [37]. Standard cylindrical sampling rings of 100 cm^3^ were taken, weighed, and dried in an oven at 105 °C until they reached a constant weight. The mass of water present in the sample is given by the difference in mass between the wet and dry sample. Water content by mass is defined as the mass of water divided by the mass of dry soil. The conversion into volumetric equivalent can be done by multiplying the gravimetric water content by the dry bulk density of the sample, with a knowledge of water density (the density of water was assumed to be 1.0 g·cm^−3^). The volumetric water content was calculated using Equation (1):

(1) $$\theta =\frac{m_{w}-m_{d}}{m_{d}}\;\rho_{b}$$

where $\rho_{b}$ (M·L^−3^) is the dry bulk density to which the material is packed, and *m_w_* (M) and *m_d_* (M) are the wet and dry material samples taken from the container.

#### 2.1.2. Instrumental Methods

##### ThetaProbe ML2x (Delta-T Devices Ltd., Cambridge, UK)

**Figure 1:**
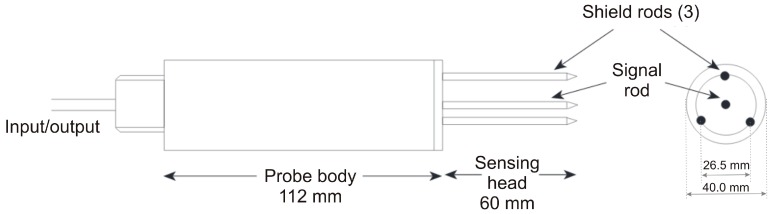
ThetaProbe scheme and dimensions [33].

ThetaProbe (Figure 1) is a commonly used impedance sensor that generates 100 MHz sinusoidal signal and outputs the measured impedance of the sampling medium as an analogue DC voltage between 0 and 1 V for a range of dielectric constant, *ε*, between 1 and 32, corresponding to a volumetric water content of approximately 0.5 m^3^·m^−3^. ThetaProbe consists of a waterproof housing containing an electronic circuit and an array of four parallel stainless steel rods (3 mm in diameter and 60 mm long). The sampling volume is defined by the dimensions of the probe: a cylinder of 40 mm in diameter and 60 mm long surrounding the centrally located signal rod (volume of approximately 75 cm^3^). The impedance of this array corresponds with the impedance of the soil, which has two components, apparent dielectric constant and ionic conductivity. The effect of ionic conductivity was minimized by employing a frequency of 100 MHz and thus the soil impedance changes depend almost solely on the apparent dielectric constant of the soil. The apparent dielectric constant of the soil is determined by the soil moisture, because the dielectric constant of water (~81) is much higher than the soil particles (3–5) and that of the air (1). The details of the measuring technique can be found in the works of Gaskin and Miller [10,12].

The relationship between ThetaProbe output (V) and square root of dielectric constant ($\sqrt{\varepsilon }$) can be fitted by a linear or third-order polynomial relationship (Equations (2) and (3)):

(2) $$\sqrt{\varepsilon }=4.44V+1.10$$

(3) $$\sqrt{\varepsilon }=4.70V^{3}-6.40V^{2}+6.40V+1.07$$

The relationship between water content *θ* and ThetaProbe voltage output *V* was defined as follows:

(4) $$\theta =\frac{\left[4.44V+1.10\right]-a_{0}}{a_{1}}$$

(5) $$\theta =\frac{\left[4.70V^{3}-6.40V^{2}+6.40V+1.07\right]-a_{0}}{a_{1}}$$

where *a*_0_ and *a*_1_ are 1.6 and 8.4, respectively, for mineral soil.

The manufacturers rated the accuracy of this generalized calibration at ±5.0% of volumetric water content [33]. To minimize the error in the generalized calibration, the manufacturer recommends using a soil-specific (own) calibration. According to the manufacturer, by executing a soil-specific calibration, the rated accuracy increases up to ±1.0% of volumetric water content *θ*. To perform a soil-specific calibration, the manufacturer recommends two different approaches: (i) a two-point technique and (ii) direct regression analysis.

(i) A two-point technique requires two raw output readings, one for the initially moist sample and the second for the dried sample (*θ* ≈ 0). Calibration coefficients *a*_0_ and *a*_1_ are determined from the wet and dry readings compared to the water contents determined by a referential (gravimetric) method. If the volumetric water content *θ* ≈ 0, the value of *a*_0_ is equal to $\sqrt{\varepsilon_{0}}$, which can be directly calculated by substituting the probe raw output from the dry sand/soil according to Equations (2) or (3). Then, after calculating the $\sqrt{\varepsilon_{w}}$ for the wet material and substituting it into the following formula together with the known values of *a*_0_ and *θ*, *a*_1_ can be obtained from the following equation:

(6) $$a_{1}=\frac{\sqrt{\varepsilon_{w}}-\sqrt{\varepsilon_{0}}}{\theta }$$

where *ε*_0_ [-] and *ε_w_* [-] are the dry and wet dielectric permittivity, respectively, and *θ* is the volumetric water content. 

With known values of *a*_1_ and *a*_0_, the volumetric water content can be calculated using Equation (4) for linear conversion and using Equation (5) for polynomial conversion of ThetaProbe output in V to $\sqrt{\varepsilon }$.

(ii) The direct regression analysis relates the raw probe output and the volumetric water content determined by the gravimetric method. Although Delta-T dataloggers are able to store a linear or non-linear conversion characteristic permanently in their software using a linearization table, an Excel spreadsheet) was used to develop the calibration equations (linear and third-order polynomial type) in this study.

##### Sensors from the ECH_2_O Family (Decagon Devices Inc., Pullman, WA, USA)

Sensors from the ECH_2_O family are widely available and use devices employing capacitance technique to determine the soil water content. In principal, the capacitance technique of soil water content determines the dielectric permittivity of the medium by measuring the charge time of a capacitor, using the soil as a dielectric medium. All sensors from the ECH_2_O family determine water content by using a dielectric measurement; the ECH_2_O EC-10 and ECH_2_O EC-20 use a measurement frequency of about 10 MHz, while ECH_2_O EC-5 and ECH_2_O TE use a measurement frequency of 70 MHz. In addition to volumetric water content, the sensor ECH_2_O TE also measures the temperature and bulk electrical conductivity. The dimensions of ECH_2_0 EC-10 are as follows: 14.5 cm (total length) × 3.17 cm (sensor width) × 0.15 cm (sensor thickness); while they are 25.4 cm × 3.17 cm × 0.15 cm for ECH_2_O EC-20. The sampling volume of the sensors ECH_2_O EC-10 and ECH_2_O EC-20 is approximately 0.7–1 cm around the sensor and runs along the length on both sides of the 10 or 20 cm long probe [24]. The manufacturer declared a 2-cm zone of influence with respect to the flat surface with very little or no sensitivity at the extreme edges of the probes [38]. Considering the dimensions, the sampling volume for ECH_2_O EC-10 can be approximated to 125 cm^3^, and 250 cm^3^ for ECH_2_O EC-20. While ECH_2_O EC-10 and ECH_2_O EC-20 differ only in the length over which the measurement is being averaged, ECH_2_O EC-5 and ECH_2_O TE were completely redesigned. ECH_2_O EC-5 and ECH_2_O TE use the same circuitry, but ECH_2_O TE has digital output instead of analog (voltage) output like the others. The dimensions of ECH_2_0 EC-5 are as follows: 8.9 cm (total length) × 1.8 cm (sensor width) × 0.7 cm (sensor thickness); while they are 10.0 cm × 3.2 cm × 0.7 cm for ECH_2_O TE. The sampling volume (maximum measurement volume) of the ECH_2_0 EC-5 and ECH_2_O TE sensors is declared by the manufacturer [39] to be 240 cm^3^ for ECH_2_0 EC-5 and 715 cm^3^ for ECH_2_O TE.

**Figure 2:**
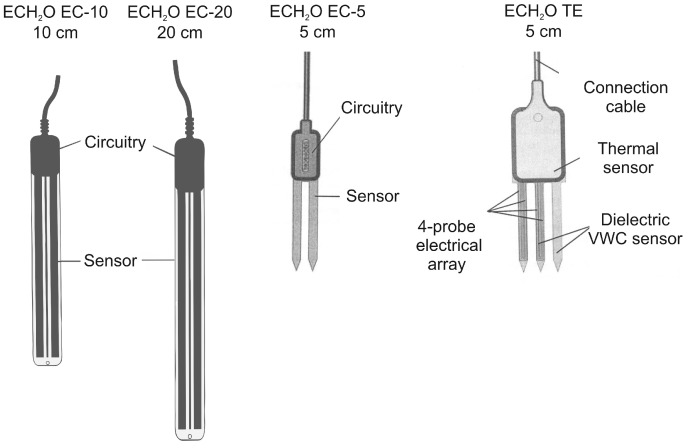
Scheme of tested soil water sensors from the ECH_2_O family (Decagon Devices Inc., Pullman, WA, USA).

The schemes and dimensions of the tested sensors from the ECH_2_O family (ECH_2_O EC-10, ECH_2_O EC-20, ECH_2_O EC-5, and ECH_2_O TE) are displayed in Figure 2.

Calibration equations for ECH_2_O EC-5, ECH_2_O EC-10, and ECH_2_O EC-20 given by the manufacturer are available in two forms: (i) applicable when a Decagon (Decagon Devices Inc., Pullman, WA, USA) datalogger is used and (ii) applicable when a non-Decagon datalogger is used. The ECH_2_O TE probe provides data in a digital form, so there is only one calibration equation for all datalogger types. Manufacturers’ calibration equations (factory calibration) used in this study with the Decagon datalogger are displayed in Table 1.

In this study, the standard calibration procedure for capacitance probes based on [40] was used. The calibration equations derived for sensors from the ECH_2_O family use a linear regression analysis of the results from gravimetrically determined volumetric water content and probe raw output readings. The simple general model is given as (Equation (7)):

(7) Y=C+mX

where *C* is a constant, intercept of the linear regression line, and m is the slope of the regression line, while *Y* is the soil volumetric water content *θ*(-) and *X* is the raw output reading of the probe (RAW). This calibration model is the one that is consistently used by the manufacturer.

### 2.2. Experimental Setup

As is indicated by the manufacturers, conducting soil-specific calibration following the recommended procedures increases the possibility of achieving better accuracy in volumetric soil water content measurements. Due to this fact and in order to compare the results from this work with the data provided by the manufacturers, precise application of the recommended procedures was maintained. In this study, both types of sensors (from the ECH_2_O family and ThetaProbe), were calibrated in laboratory conditions with a constant temperature of 20 °C for two porous materials; (i) silica sand and (ii) loess. The silica sand (produced by Sklopísek Střeleč, a.s., Czech Republic) had a high content of SiO_2_ (about 98.6%) and its particle size ranged from 0.063 to 0.400 mm with a mean diameter of 0.14 mm. The particle density of 2.65 g·cm^−3^ was determined by the water pycnometer method. The loess (Haplic Chernozem, substrate loess) was taken from the experimental field of the Czech University of Life Sciences, Prague, Czech Republic. The field is located at an altitude of 286 m above sea level with latitude 50°08′ N and longitude 14°24′ E. The average surface dry bulk density measured at 5-cm intervals up to 20 cm depth varied from 1.3 to 1.5 g·cm^−3^ with average particle density of 2.5 g·cm^−3^ [41].

The sieved material (particles smaller than 2 mm in diameter) was packed into a calibration container of rectangular shape with dimensions of 33 cm (length) × 23 cm (width) × 13 cm (height) with a volume of 10 L. The sand/soil was packed as homogeneously throughout the container as possible. The sand/soil was packed layer by layer in eight layers; each layer, about 1.6 cm thick, was packed and compacted into its defined volume and dry bulk density before adding the next one. The procedure to create rather homogeneous dry bulk density of sand/soil packing is almost identical to the procedure published several times in the scientific literature, for example in [23]. For sand, three different levels of dry bulk density were prepared for each tested sensor; (i) loose; (ii) moderate; and (iii) compact packing. (i) Loose packing (LP) for sensors from the ECH_2_O family had an average dry bulk density of 1.00 g·cm^−3^ and 1.16 g·cm^−3^ for testing the ThetaProbe; (ii) Moderate packing (MP) for sensors from the ECH_2_O family had an average dry bulk density of 1.20 g·cm^−3^ and 1.35 g·cm^−3^ for testing of the ThetaProbe; (iii) Compact packing (CP) for sensors from the ECH_2_O family had an average dry bulk density of 1.40 g·cm^−3^ and 1.62 g·cm^−3^ for testing of the ThetaProbe. Two 10-L containers (see above) were prepared for each tested soil water content. Sensors from the ECH_2_O family were placed together into one container, while ThetaProbe was placed in the other one. Unfortunately, the same values of dry bulk densities were not reached in the second container where the ThetaProbe was tested. This fact made a direct comparison with the ECH_2_O sensors more difficult; however, three levels (LP, MP and CP) were observed. Attention was paid to the homogeneity of the bulk density evaluated, for comparison on three cores (applied classical “core method” for determination of dry bulk density) taken from a box of certain bulk density at the end of each sensor test (see again [23]). For the Chernozem soil, only one level of dry bulk density was prepared and evaluated, an average of 1.20 g·cm^−3^. This value was difficult to obtain for higher soil water contents, namely 29% and 39% by volume, when values of dry bulk density reached 1.30 and 1.35 g·cm^−3^; the dry bulk density referred to in the remainder of the text as moderate packing (MP).

When the measurement was finished an undisturbed soil sample (100 cm^3^) was taken from the surface at each soil water content step to check the soil water content and dry bulk density of each container. The sensors ECH_2_O EC-5, ECH_2_O TE, and ThetaProbe were inserted vertically from the surface into the filled container, while sensors ECH_2_O EC-10 and ECH_2_O EC-20 were fully buried horizontally. The insertion angle was thus an issue of difference. However, the effect of the insertion angle was tested using sand with a constant dry bulk density (CP with a value of 1.5 g·cm^−3^) and water content (15.0 vol %). No statistically significant difference was observed for insertion angle (vertical, horizontal) for each of the tested sensors (SD ranged from 0.0 to 0.68 mV for vertical installation and from 0.0 to 0.90 mV for horizontal installation). The main source of non-unified results can be attributed to the achieved level of homogeneity rather than to the insertion angle.

Special attention was paid to the sensor placement within the container; sensor dimensions, sampling volumes, and measurement ranges were taken into account to ensure unaffected measurements. A plastic spoon was used to dig a shallow trench in the half-full soil container for a full length of ECH_2_O EC-10 and ECH_2_O EC-20 sensors. The sensors were placed in the same depth (on the fourth soil/sand layer) next to each other, 5 cm from the plastic side of the container. Each sensor was set into its own trench with its long axis horizontally located and the flat plane of the probe oriented vertically. A small amount of sand or soil material was added over the probes and evenly packed on both sides of the probes. The same procedure was repeated until the probe was completely buried under the sand/soil material with the desired dry bulk density. Then the container was topped up again in layers. Then the ECH_2_O EC-5 and ECH_2_O TE sensors were fully inserted from the surface; there was a 3 cm thick soil/sand layer between the edges of the ECH_2_O EC-5 and ECH_2_O TE prongs and depth with plates of the ECH_2_O EC-10 and ECH_2_O EC-20 sensors (considering the 2-cm range of ECH_2_O EC-10 and ECH_2_O EC-20 sensors, 1 cm of maximal reach from the prongs’ tips, and the 3 cm soil/sand layer) [38,39]. Each sensor was also 5 cm from the plastic side of the container and 10 cm from each other [39].

This packing procedure was repeated for eight different water contents for sensors from ECH_2_O family and for nine different water contents for testing the ThetaProbe for each type of packing. The standard method, which is recommended by Decagon Devices Inc. in their calibration procedure, was used. The soil moisture was changed in one direction only by step-by-step wetting and uniformly mixing throughout until it almost reached saturation, so the effect of the hysteresis could be neglected. After the probes were inserted, readings were taken with an Em50 datalogger (Decagon Devices, Inc., Pullman, WA, USA) for sensors from the ECH_2_O family and an HH2 moisture meter (Delta-T Devices Ltd., Cambridge, UK) for ThetaProbe. The data were collected as unprocessed output, raw reading, or voltage in mV and as volumetric water content in %, which was used in the quality evaluation of the factory calibration.

The performance of the probes was also studied with respect to their sensitivity to changes in the electrical conductivity of the sand. A solution prepared by dissolving 2 g of Sodium Chloride (NaCl) into distilled water was used to wet the silica sand packed to THE desired dry bulk density of (1.35 g·cm^−3^). The silica sand was wetted from its air dry water content up to near saturation and 6 to 7 points of soil water measurements were taken for different levels of water content. Then the same soil was allowed to air dry again, so the other set of measurements following the same wetting procedure was evaluating the probes’ performance for more saline conditions. This procedure was repeated one more time to obtain three levels of sand salinity.

### 2.3. Sensor Performance Evaluation

The sensor performance was evaluated mainly with respect to accuracy, meaning how close the measured values of soil water content were in comparison with the “true” values determined by the reference method (volumetric water content based on the determination by the gravimetric method).

#### 2.3.1. Absolute Difference (AD)

In the literature [42], the absolute difference between two real numbers is defined as the distance on the real line between these two points. Doležal et al. [22] used the absolute difference for evaluation of measurement accuracy of ThetaProbe sensors. The absolute difference can be described by the following equation:

(12) $$AD=\left|\theta_{real}-\theta_{measured}\right|$$

where *θ_real_* is the volumetric water content determined by multiplying the dry bulk density by the gravimetric sand/soil water content and *θ_measured_* is the volumetric water content determined by the particular soil water sensor based on the factory and our own calibration equations.

#### 2.3.2. Standard Deviation (SD)

Standard deviation is one of the most used measures quantifying the amount of variation of a set of data, describing how tightly all the data are clustered around the mean of the dataset. Its value is strongly influenced by outlying values [43]. Standard deviation is expressed in the same units as the data being evaluated. A standard deviation close to 0 indicates that the data points tend to be very close to the mean value of the dataset, while a high value of standard deviation indicates that the data points are spread out over a wide range of values. The standard deviation of a sample of the population can be calculated as follows:

(13) $$SD=\sqrt{\frac{\sum {\left(\theta -\bar{\theta }\right)}^{2}}{n-1}}$$

where *θ* is the water content under an evaluation, $\bar{\theta }$ is the mean value of the data set (water content under evaluation), and *n* is the number of measurement points.

#### 2.3.3. Root Mean Square Error (RMSE)

Root mean square error or root mean square deviation is a frequently used measure of the difference between the value predicted by a model and the “real” value actually observed. The root mean square error represents the sample standard deviation of the differences between predicted and observed values. The root mean square error was used as a measure of accuracy in different published works dealing with soil water content determination [3,8,44]. Root mean square error is a good measure of accuracy when comparing different models to predict only a particular variable, because it is a scale-dependent measure. Root mean square error can be calculated using Equation (14):

(14) $$RMSE=\sqrt{\frac{1}{n}\sum {\left(\theta_{real}-\theta_{measured}\right)}^{2}}$$

where *θ_real_* is the volumetric water content determined by multiplying the dry bulk density by the gravimetric sand/soil water content, *θ_measured_* is the volumetric water content determined by the particular soil water sensor based on the factory and our own calibration equations, and *n* is the number of measurement points.

#### 2.3.4. Coefficient of Determination

Coefficient of determination is a measure used in statistical model analysis to evaluate how well a model explains and/or predicts future outcomes. It is used as an accuracy indicator for the applied model. In general, a model fits the data well if the differences between the observed values and the values predicted by a model are small and unbiased. The coefficient of determination can be defined as the percentage of the response variable variation that is explained by a model; its value in percentage is always between 0 and 100. In general, the higher the value the better the model fits the data; however, it cannot determine whether the coefficient estimates and predictions are biased [45]. The coefficient of determination is usually denoted as *R*^2^, *r*^2^, or *R*-square, referring to the squared value of correlation coefficient, *r*. The correlation coefficient measures the strength and direction of a relationship (linear) between two variables. The value of the correlation coefficient is between −1 and +1; the + sign is used for positive linear correlation, while the – sign is used for negative correlation. If there is not any or a weak correlation, the value of *r* is close to 0.

The mathematical formula for computing the correlation coefficient is as follows:

(15) $$r=\frac{N\sum_{i=1}^{N}x_{i}y_{i}-\sum_{i=1}^{N}x_{i}\sum_{i=1}^{N}y_{i}}{\sqrt{\left[N\sum_{i=1}^{N}x_{i}{}^{2}-{\left(\sum_{i=1}^{N}x_{i}\right)}^{2}\right]\left[N\sum_{i=1}^{N}y_{i}{}^{2}-{\left(\sum_{i=1}^{N}y_{i}\right)}^{2}\right]}}$$

where *x_i_* is an independent variable, *y_i_* is a dependent variable, and *N* denotes the number of data pairs *x_i_* and *y_i_*.

#### 2.3.5. Analysis of Variance (ANOVA)

Analysis of variance is a particular form of statistical hypothesis testing used in the analysis of experimentally observed data. It can be used as an explanatory tool to explain observations. The calculations of ANOVA can be characterized as computing a number of means and variances, dividing two variances and comparing the ratio to a “true” value to determine statistical significance. The effect of any treatment is estimated by taking the difference between the mean of the observations that receive the treatment and the general mean [46]. Analysis of variance is usually performed in order to confirm a statistically significant difference between groups on some variable. Analysis of variance on significance level α = 0.05 for RMSE values of soil water contents determined by gravimetric method and by tested sensor application was carried out in this study.

## 3. Results

### 3.1. Results for ThetaProbe ML2x (Delta-T Devices Ltd., Cambridge, UK)

The soil-specific calibration of the ThetaProbe was carried out for sand/soil material by both of the above described approaches: (i) a two-point technique and (ii) direct regression analysis. The factory and our own values of calibration constants *a*_0_ and *a*_1_ are described in the table below (Table 2). The possible range of parameters *a*_0_ and *a*_1_ given by the manufacturer for mineral soils is between 1 and 2 for *a*_0_ and between 7.6 and 8.6 for *a*_1_. Just on the basis of comparisons of obtained values of *a*_0_ and *a*_1_ for our own linear and our own polynomial calibrations for sand/soil material, no significant improvement of soil water determination was expected. Some of the values of *a*_1_ were outside of the commonly obtained range of values. This is supported by higher values of RMSE for most of the water content data based on our own two-point calibrations (Table 3).

**Figure 3:**
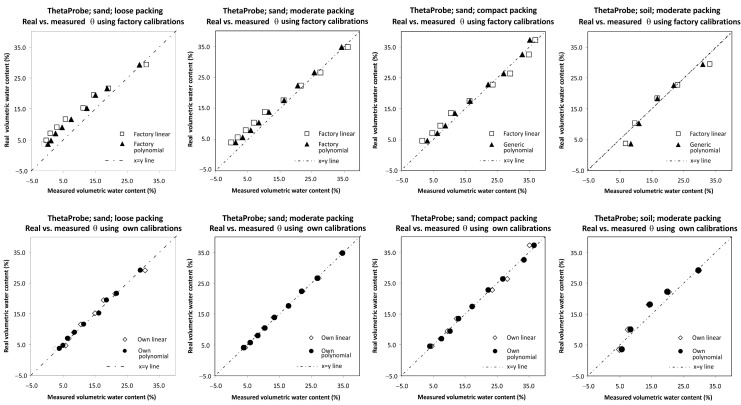
Graphical comparison of the real volumetric water contents obtained on the basis of the gravimetric method and volumetric water contents measured by ThetaProbe using factory and our own linear and polynomial calibration equations.

Based on the results, the determination of own parameters for the two-point calibration approach cannot be recommended for sand/soil materials tested in this study, as slightly less accurate results of soil water content measurement were obtained in comparison with the simple use of factory calibration settings. This result is in agreement with studies [11,47] that concluded that the difficulty especially in sandy soil originates in the pore system’s instability in dry conditions and thus affects the *a*_0_ determination. On the contrary, the second approach (obtaining our own calibration equations by direct regression analysis) provided better results than those based on the factory calibration settings. Our own linear and third-order polynomial equations are listed in Table 4. The better accuracy obtained by application of our own linear and third-order polynomial equations is confirmed by the lower values of AD and RMSE (Table 5). A graphical comparison of the real volumetric water contents obtained on the basis of the gravimetric method and the volumetric water contents measured by ThetaProbe using factory and our own linear and polynomial calibration equations described in Table 4 is presented in Figure 3.

### 3.2. Results for Sensors from the ECH_2_O Family (Decagon Devices Inc., Pullman, WA, USA)

The soil-specific calibration of the four selected sensors from the ECH_2_O family (ECH_2_O EC-10, ECH_2_O EC-20, ECH_2_O EC-5, and ECH_2_O TE) was carried out for sand/soil material using the direct regression approach. Our own linear calibration equations for loose, moderate, and compact packing of sand and moderate packing of soil are displayed in Table 6.

**Figure 4:**
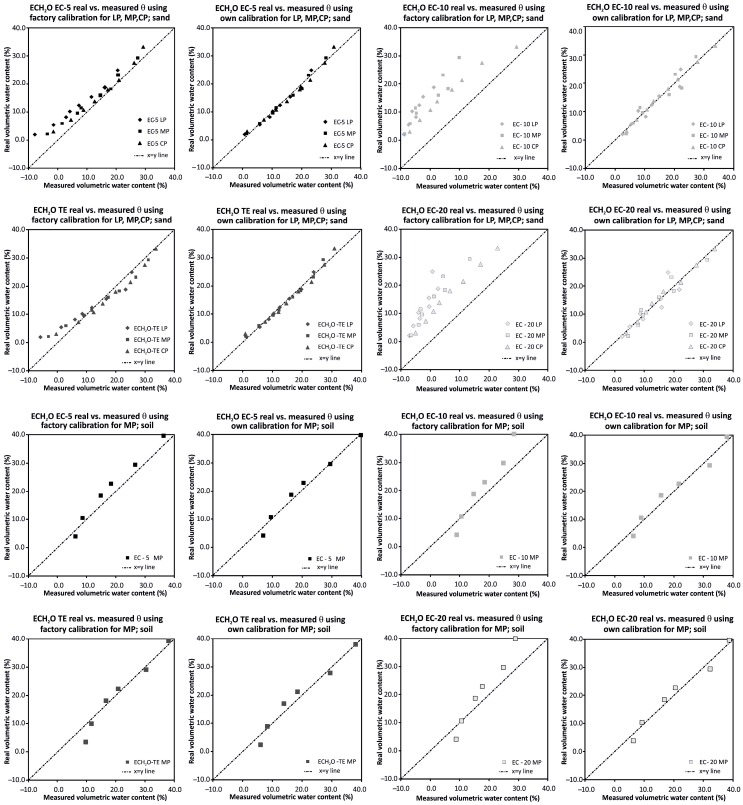
Graphical comparison of the real volumetric water contents obtained on the basis of gravimetric method and volumetric water contents measured by the sensors from the ECH_2_O family using factory and our own linear calibration equations.

The ability of the sensor to provide an accurate soil water content measurement was described graphically (Figure 4) and evaluated by the same statistical measures as ThetaProbe; determination coefficient *R*^2^, absolute difference AD (vol %), and RMSE (vol %). These statistics are summarized in Table 7 and discussed later on.

### 3.3. Performance Evaluation of Tested Sensors to Measure the Water Content in Saline Conditions

The performance of all tested sensors was also evaluated for their possible use in the field with saline irrigation water. The test was carried out for sandy medium of a dry bulk density of 1.35 g·cm^−3^ (compact packing). The saline irrigation water was prepared as a solution of 2 g of table salt (NaCl) in 1.0 L of distilled water, creating a solution with electrical conductivity (EC) of 3300 mS·m^−1^. The sand was wetted by the salt solution step by step in six or seven stages from dry to ca. 25 vol %. After the measurements at the last stage the sand was dried (the water evaporated while the salt remained) and another wetting procedure with the salt solution started. In total, three rounds of drying/wetting of the sand were carried out, simulating the repeated use of saline water for irrigation. A set of reference data based on non-saline water application was also prepared (salinity 0) and volumetric water contents were used for the “Reference line” construction. Factory linear calibrations (Equation (8) for ThetaProbe and equations displayed in Table 1 for sensors from the ECH_2_O family) and our own linear calibration equations for sandy material under compact packing (equations displayed in Table 4 for ThetaProbe and in Table 6 for sensors from the ECH_2_O family) were applied to evaluate the ability of the sensor to accurately measure the volumetric water content in saline conditions. Both factory and our own linear calibrations performed quite well for all tested sensors for the first round of the drying/wetting cycle. The more saline the conditions, the higher the AD between the measured and real values of volumetric water contents. Furthermore, with increasing salinity (after the second and third rounds of the drying/wetting cycle), the sensors ECH_2_O EC-10 and ECH_2_O EC-20 performed poorly, consistently and significantly overestimating the real volumetric water contents. For the first round of the drying/wetting cycle, the averaged AD ranged between 0.64 vol % (ECH_2_O TE) and 4.09 vol % (ECH_2_O EC-20). For the second and third rounds the ADs were much bigger for sensors ECH_2_O EC-10 and ECH_2_O EC-20 (average AD ranged between 10.36 vol % and 16.74 vol %) when compared to the other three tested sensors, where the average AD ranged between 1.75 vol % and 16.74 vol %. Our own linear calibrations performed quite well only for three out of the five tested sensors. ECH_2_O EC-10 and ECH_2_O EC-20 practically failed to measure the volumetric water content in saline conditions (after the second round of wetting by saline water). The AD between the real volumetric water contents and those measured by the sensors ECH_2_O EC-10 and ECH_2_O EC-20 increased significantly after the second and again after the third round of the drying/wetting cycle (the average AD started at about 12 vol %, than increased up to about 21 vol %, and for the third round the values reached about 27 vol %). Detailed AD and RMSE values for all five tested sensors for factory and our own linear calibrations are presented in Table 8. Probably the best and most stable measurements over the range of all three rounds of drying/wetting cycles with saline water application was provided by ECH_2_O EC-5, regardless of the calibration type (linear or our own). Also, ThetaProbe provided very stable and linearly related results with slightly higher AD than ECH_2_O EC-5 for more saline conditions (after the second and third rounds of the wetting/drying cycle). However, the accuracy of the measurements was within an acceptable range for the first and second rounds of the wetting/drying cycle (RMSE < 5 vol %). The accuracy can be improved in more saline conditions by using salinity-specific calibration because of the stable and linear outcome when relating measured and real volumetric water contents, which were consistently overestimated.

**Figure 5:**
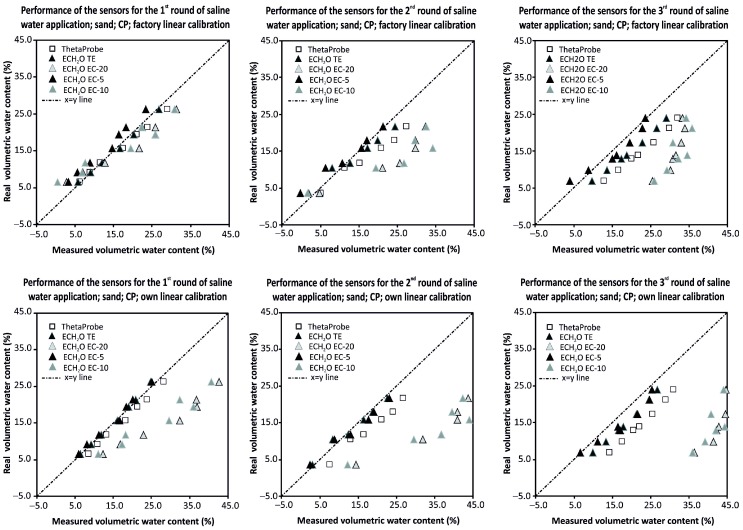
Performance of all tested sensors for three rounds of saline water applications for a sand media and compact packing using factory and our own linear calibration equations.

A graphical comparison of the ability of the tested sensors to measure the volumetric water content under saline conditions using factory and own linear calibrations is given in Figure 5.

**Figure 6:**
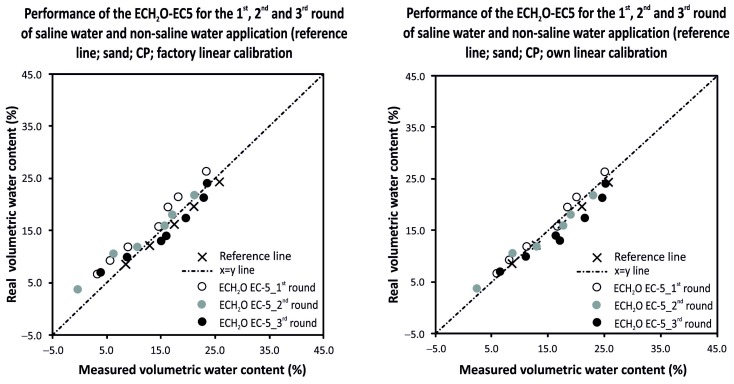
Real and measured volumetric water contents determined by ECH_2_O EC-5 for all three rounds of the drying/wetting cycle with saline water application with an indication of a reference line that was based on non-saline water application.

**Figure 7:**
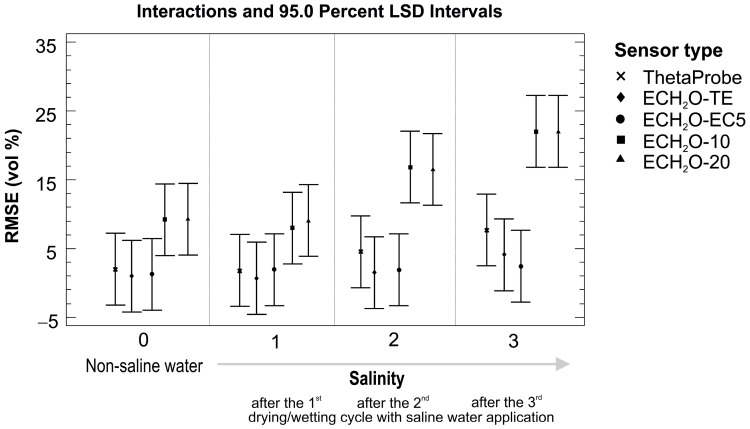
Sensor performance comparison based on RMSE (vol %) values for saline conditions.

Analysis of variance based on the RMSE values for measured and real volumetric water contents confirmed some expectations and also revealed some surprising results. The five tested sensors were grouped into two homogeneous groups that performed significantly differently from each other. Good performance was provided by sensors from the ECH_2_O family working on higher frequency (ECH_2_O EC-5, ECH_2_O TE (LS mean cca 1.8 vol %) and ThetaProbe (LS mean cca 4.0 vol %)) but worse performance was seen for the ECH_2_O sensors working at lower frequencies (ECH_2_O EC-10 and ECH_2_O EC-20 (LS mean cca 14.1 vol %)). As expected, the higher the salt content, the higher the value of RMSE; however, ECH_2_O EC-5 also performed very well after the third round of the drying/wetting cycle, not differing much from the reference line when non-saline water was applied (Figure 6 and Figure 7). Figure 7 also presents a statistical comparison of ECH_2_O EC-5 with other four tested sensors, showing significantly higher RMSE values for measurements with ECH_2_O EC-10 and ECH_2_O EC-20.

## 4. Discussion

### 4.1. Discussion of the Results of ThetaProbe ML2x

**Figure 8:**
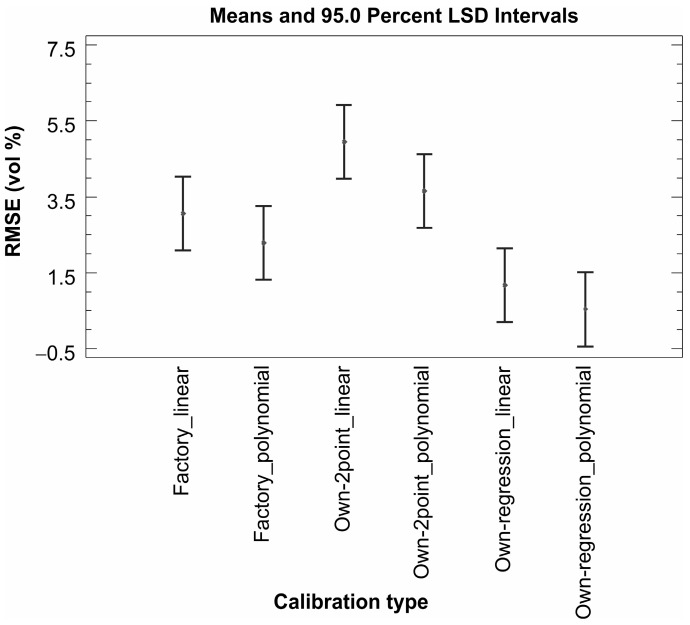
ThetaProbe—means and 95.0% LSD intervals of analysis of variance for RMSE and calibration type.

**Figure 9:**
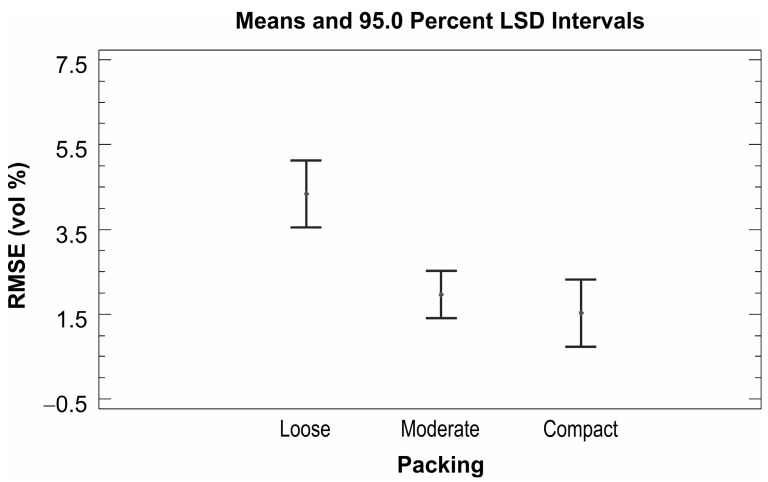
ThetaProbe—means and 95.0% LSD intervals of analysis of variance for RMSE and packing.

The analysis of variance showed a statistically significant relationship between RMSE and both predictor variables (calibration type and packing) at the 95.0% confidence level. The predictor material was removed from the model as it was shown that this predictor does not have a statistically significant relationship with the RMSE. Practically no statistically significant differences were obtained when all types of ThetaProbe calibration were compared (Figure 8); however, some improvement in accuracy was obtained when our own linear and third-order polynomial calibrations were determined using the direct regression approach. The effect of dry bulk density factor (packing) shows the importance of good contact between the probe sensing rods and soil/sand particles, because significantly higher RMSE values were obtained for loose packing (Figure 9). All the results indicate the need for our own calibration (direct regression approach) for sand/soil with lower values of dry bulk density.

Recently, wireless sensor network technology has been used for catchment-scale measurements and hydrological processes descriptions, e.g., [48,49]. According to [11], ThetaProbe has received acceptance in the remote sensing community for soil water content measurements at the soil surface [50]. The field soil-specific calibrations based on the direct regression approach can lead to more precise outcomes and thus justify its labor and cost.

### 4.2. Discussion of the Results for Sensors from the ECH_2_O Family

**Figure 10:**
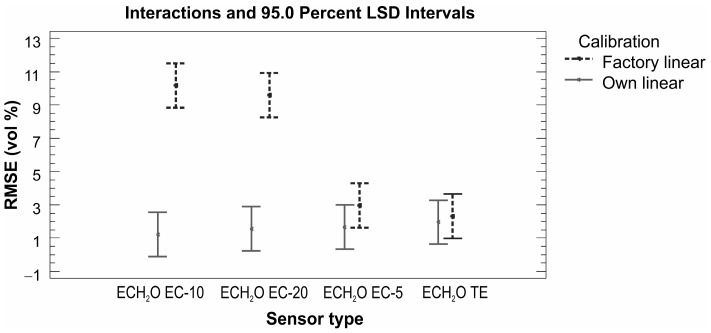
Sensors from the ECH_2_O family—sensor performance comparison based on RMSE (vol %) values for factory and our own calibrations.

**Figure 11:**
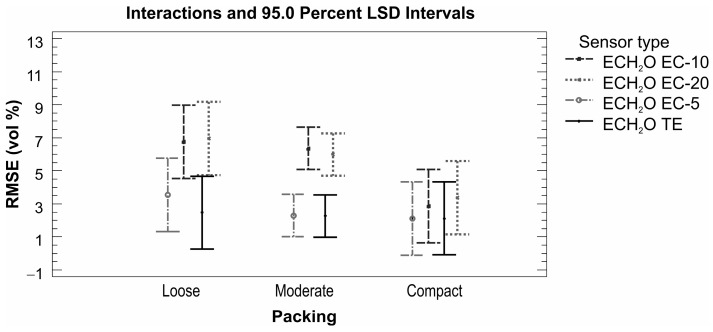
Sensors from the ECH_2_O family—sensor performance comparison based on RMSE (vol %) values for different dry bulk density levels.

When evaluating the performance of sensors from the ECH_2_O family, it is necessary to mention the fact that the sensors have different sizes, shapes, and operating frequencies. The first two factors are suppressed by the used homogeneous material placed in a large rectangular container of a volume of 10 L. According to the applied operating frequency, the sensors from the ECH_2_O family can be divided into two groups: (i) sensors operating at a frequency of 70 MHz (ECH_2_O TE and ECH_2_O EC-5) and (ii) sensors operating at a frequency of 5 MHz (ECH_2_O EC-10 and ECH_2_O EC-20). The same groupings are reflected in the evaluation of measurement accuracy: sensors operating at a higher frequency performed significantly better (average RMSE 3.3 vol % for factory calibration and 1.3 vol % for our own linear calibration; see Table 7). The factory calibrated sensors used in loosely packed sand consistently undermeasured the values of volumetric water content when compared to those determined on the basis of the gravimetric method. This was particularly evident for sensors ECH_2_O EC-10 and ECH_2_O EC-20, where the absolute differences between the measured and real values ranged between 1.3 and 13.1 vol % (Table 7). For compacted sand the maximum value of average absolute difference decreased to 7.9 vol %, which is still too high for accurate measurement (Table 7). This finding is in agreement with the manufacturer’s statement that the factory calibrations for ECH_2_O EC-10 and ECH_2_O EC-20 sensors do not provide sufficiently accurate results and an own calibration is needed. The quality of the own calibration performance is confirmed by the results obtained in this study (Figure 10). This is in accordance with the results of Kizito et al. [20], who concluded that the sensitivity of the capacitance sensors’ measurements is primarily affected by their measurement frequency. A statistically significant effect of the material (sand/soil) in which the volumetric water content was measured was observed for sensors operating at a lower measurement frequency of 5 MHz (ECH_2_O EC-10 and ECH_2_O EC-20). Regardless of the applied calibration type, the dry bulk density effect appeared to be significant, especially for the ECH_2_O EC-10 and ECH_2_O EC-20 sensors (Figure 11). Similarly to the ThetaProbe, the results show the importance of good contact between the sensors and sand/soil being measured, which the compact packing ensures.

A significant improvement in recently developed sensors from the ECH_2_O family ECH_2_O EC-5 and ECH_2_O TE was observed in this study. In comparison with the older ECH_2_O EC-10 and ECH_2_O EC-20, they are able to measure volumetric water content with good accuracy regardless of the soil type and dry bulk density value. Naturally, if a soil water content sensor is to reach its maximum accuracy, a soil-specific calibration needs to be carried out [51,52].

On the contrary, specifically calibrated older sensors ECH_2_O EC-10 and ECH_2_O EC-20 can perform measurements with good accuracy of a more representative value as higher volumes of soil/sand are being sampled and averaged to provide volumetric water content reading. Czarnomski et al. [21], on the basis of a field experiment, concluded that ECH_2_O EC-20 (in the text designated as EC-20) performed nearly as well as a TDR probe, which is commonly assumed to be one of the most accurate water content probes.

### 4.3. Discussion of the Ability of Tested Sensors to Measure the Water Content in Saline Conditions

**Figure 12:**
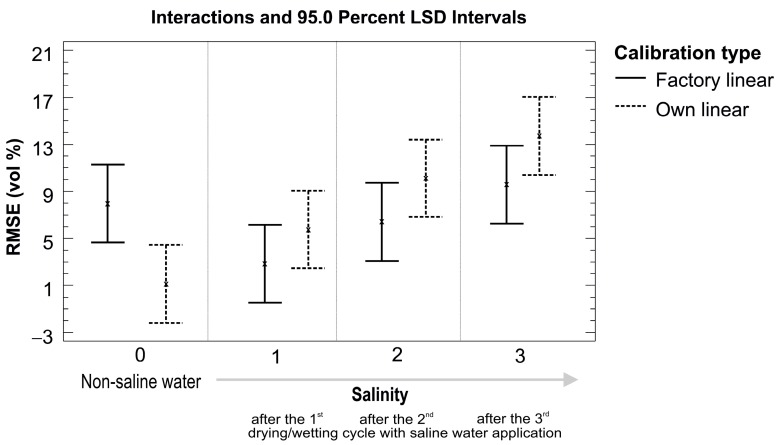
Factory and our own calibration comparison based on RMSE (vol %) values for saline conditions.

Testing of the sensors under saline conditions was performed in three rounds, as described in Section 2.2 and Section 3.3. Usually, the use of our own calibration equations for non-saline conditions means a significant improvement in the measurement accuracy of the tested sensors. Surprisingly, this statement is not valid for saline conditions, where, in general, factory linear calibrations consistently performed better than our own calibrations for all three rounds of the drying/wetting cycle with saline water application (Figure 12).

Although the differences were not statistically significant for each drying/wetting cycle of saline water application, the statistical results need to be checked for individual sensors, as there are big differences between them (Table 8). The own linear calibration for ECH_2_O TE performed better than the factory linear calibration for all three drying/wetting cycles, while our own calibration for ECH_2_O EC-5 only did so for the first two cycles. The differences between the RMSE values (factory vs. own) ranged from 0.11 to 1.19 vol % for ECH_2_O TE and from −0.94 to 1.95 vol % for ECH_2_O EC-5. ThetaProbe performed better with the factory linear calibrations for all three drying/wetting cycles; the differences between the RMSE values (factory vs. own) ranged from −0.07 to −0.48 vol % for ECH_2_O TE. The ECH_2_O EC-10 and ECH_2_O EC-20 performed similarly, with poor accuracy in saline conditions. The values of RMSE (vol %) increased with increasing salinity, on average from 4.5 vol % to 16.8 vol % for factory linear calibration and from 12.6 vol % to 27.2 vol % for our own linear calibration for non-saline conditions. These high values affected the statistical outcome when evaluating all sensors together in Figure 12, showing the factory linear calibration as a better choice when compared to the own linear calibration.

The findings of this study are in accordance with Seyfried and Murdock [18], who tested the adapted ECH_2_O TE and ECH_2_O EC-5 sensors for different measurement frequencies. The sensitivity of the tested sensors to given levels of salinity decreased with increasing operating frequency. The differences in accuracy of soil water content measurement between the tested sensors from the ECH_2_O family can be explained mainly by the differences in their operating frequency. Results obtained by Bogena et al. [3], who tested the ECH_2_O EC-5 sensor, showed its sensitivity to bulk electrical conductivity with a maximum error of 6 vol % for bulk electrical conductivity of 1 dS·m^−1^ and soil water content of 51 vol %. They also stated that the older type ECH_2_O EC-20 was more sensitive to bulk electrical conductivity than ECH_2_O EC-5. Scudiero et al. [53] suggested using a newer version of ECH_2_O TE, ECH_2_O 5TE, which works at a frequency of 70 MHz for continuous monitoring of soil moisture content and solute dynamics in saline soils. 

## 5. Conclusions

The final selection and application of the particular soil water content sensor is based on a combination of different factors that need to be taken into account: suitability of the sensor for the particular purpose (material type, measured volume, sensor durability, etc.), the required accuracy of the measurement, and, last but not least, the cost. The published experience of others can help with this decision-making process. Research on soil water content measurement is a dynamic process reflecting the need for relatively cheap, precise, and automated systems, especially for irrigation purposes, where saving water has become a very pressing issue.

This study evaluated five commercially available and financially affordable soil water content sensors. All five tested sensors (ThetaProbe ML2x produced by Delta-T Devices, Ltd., Cambridge, UK, and sensors from the ECH_2_O family produced by Decagon Devices Inc., Pullman, WA, USA: ECH_2_O EC-10, ECH_2_O EC-20, ECH_2_O EC-5, and ECH_2_O TE) performed very well if the proper calibration was applied. The two-point calibration approach for ThetaProbe did not lead to any measurement accuracy improvement and thus cannot be recommended. On the contrary, application of the direct regression approach has led to an improvement in accuracy for all five tested sensors. Generally, the measurement accuracy increased with increasing value of dry bulk density, emphasizing the importance of good contact between the sensor and the sand/soil. For use in saline conditions, only three out of the five tested sensors can be recommended: ECH_2_O EC-5, ECH_2_O TE, and ThetaProbe. For ThetaProbe applications in saline conditions, when only factory and our own calibrations for non-saline conditions are available, factory calibration leads to more accurate results.

## Figures and Tables

**Table 1:** Factory calibration equations for sensors from the ECH_2_O family using Decagon datalogger (Equations (8)–(11)).

Sensor Type	Calibration Equation	Equation Number
ECH_2_O TE	*θ* = 10.90 × 10^−4^ *RAW* − 0.629	(8)
ECH_2_O EC-5	*θ* = 8.50 × 10^−4^ *RAW* − 0.480	(9)
ECH_2_O EC-10	*θ* = 5.71 × 10^−4^ *RAW* − 0.376	(10)
ECH_2_O EC-20	*θ =* 4.24 × 10^−4^ *RAW* − 0.290	(11)

**Table 2:** Values of factory and own constants *a*_0_ and *a*_1_ for two-point ThetaProbe calibration equations for sand/soil materials.

Type of Conversion	Constants	Loose Packing (Sand)	Moderate Packing (Sand)	Compact Packing (Sand)	Moderate Packing (Soil)
Linear conversion of V output to $\sqrt{\varepsilon }$	*a*_0_	1.46	1.47	1.49	1.72
	*a*_1_	5.29	7.06	8.1	9.02
Polynomial conversion of V output to $\sqrt{\varepsilon }$	*a*_0_	1.54	1.56	1.58	1.85
	*a*_1_	5.34	7.02	7.88	7.96
Factory settings for mineral soils	*a*_0_	1.6			
	*a*_1_	8.4			

**Table 3:** Performance comparison of factory and own calibrations for two-point calibration approach of ThetaProbe: *R*^2^ values, average AD (vol %), and RMSE values (vol %).

Calibration Type	Tested Material	Packing	*R*^2^	Average AD (vol %)	RMSE (vol %)
Linear factory	Soil	Moderate	0.953	1.88	2.32
Polynomial factory	Soil	Moderate	0.949	1.89	2.45
Linear own	Soil	Moderate	0.953	2.05	2.42
Polynomial own	Soil	Moderate	0.949	2.03	2.36
Linear factory	Sand	Loose	0.982	4.51	4.73
Polynomial factory	Sand	Loose	0.991	3.40	3.61
Linear own	Sand	Loose	0.982	6.17	9.06
Polynomial own	Sand	Loose	0.991	4.98	7.08
Linear factory	Sand	Moderate	0.998	2.64	2.95
Polynomial factory	Sand	Moderate	0.999	1.77	1.91
Linear own	Sand	Moderate	0.998	3.64	4.89
Polynomial own	Sand	Moderate	0.999	2.34	3.14
Linear factory	Sand	Compact	0.990	2.35	2.17
Polynomial factory	Sand	Compact	0.995	1.21	1.16
Linear own	Sand	Compact	0.990	3.01	2.78
Polynomial own	Sand	Compact	0.995	1.36	1.36

**Table 4:** Own linear and third-order polynomial calibration equations for ThetaProbe (direct regression approach).

Tested Material	Packing	Regression Type	Equation
Soil	Moderate	Linear	*y* = 49.734*x* − 5.672
Soil	Moderate	Third-order polynomial	*y* = 360.14*x*^3^ − 596.55*x*^2^ + 355.24*x* − 52.53
Sand	Loose	Linear	*y* = 41.925*x* + 1.329
Sand	Loose	Third-order polynomial	*y* = 63.827*x*^3^ − 101.39*x*^2^ + 86.101*x* − 3.236
Sand	Moderate	Linear	*y* = 43.703*x* − 0.421
Sand	Moderate	Third-order polynomial	*y* = 43.165*x*^3^ − 59.145*x*^2^ + 66.790*x* − 2.788
Sand	Compact	Linear	*y* = 45.577*x* − 1.886
Sand	Compact	Third-order polynomial	*y* = 115.52*x*^3^ − 155.39*x*^2^ + 105.12*x* − 8.1418

**Table 5:** Performance comparison of factory (displayed in Table 3) and our own calibrations for direct regression approach of ThetaProbe: *R*^2^ values, averaged AD (vol %), and RMSE values (vol %).

Calibration Type	Tested Material	Packing	*R*^2^	Average AD (vol %)	RMSE (vol %)
Linear own	Soil	Moderate	0.953	1.78	1.46
Polynomial own	Soil	Moderate	0.999	0.19	0.16
Linear own	Sand	Loose	0.982	0.92	1.08
Polynomial own	Sand	Loose	0.997	0.35	0.47
Linear own	Sand	Moderate	0.998	0.34	0.41
Polynomial own	Sand	Moderate	0.999	0.22	0.24
Linear own	Sand	Compact	0.990	0.79	1.10
Polynomial own	Sand	Compact	0.997	0.59	0.62

**Table 6:** Our own linear calibration equations for sensors from the ECH_2_O family (RAW is the sensor output in mV and *θ* is water content in vol %).

Sensor Type	Material	Packing	Calibration Equation
ECH_2_O TE	Soil	Moderate	*θ* = 0.1248 *RAW* − 76.164
ECH_2_O EC-5	Soil	Moderate	*θ* = 0.0949 *RAW* − 53.587
ECH_2_O EC-10	Soil	Moderate	*θ* = 0.0906 *RAW* − 66.463
ECH_2_O EC-20	Soil	Moderate	*θ* = 0.0686 *RAW* − 54.38
ECH_2_O TE	Sand	Loose	*θ* = 0.0689 *RAW* − 34.96
ECH_2_O EC-5	Sand	Loose	*θ* = 0.0671 *RAW* − 31.113
ECH_2_O EC-10	Sand	Loose	*θ* = 0.1097 *RAW* − 52.722
ECH_2_O EC-20	Sand	Loose	*θ* = 0.0836 *RAW* − 40.528
ECH_2_O TE	Sand	Moderate	*θ* = 0.0831 *RAW* − 44.811
ECH_2_O EC-5	Sand	Moderate	*θ* = 0.0734 *RAW* − 37.168
ECH_2_O EC-10	Sand	Moderate	*θ* = 0.0722 *RAW* − 32.798
ECH_2_O EC-20	Sand	Moderate	*θ* = 0.0567 *RAW* − 25.485
ECH_2_O TE	Sand	Compact	*θ* = 0.0951 *RAW* − 53.7
ECH_2_O EC-5	Sand	Compact	*θ* = 0.0815 *RAW* − 43.313
ECH_2_O EC-10	Sand	Compact	*θ* = 0.0557 *RAW* − 26.018
ECH_2_O EC-20	Sand	Compact	*θ* = 0.0450 *RAW* − 21.359

**Table 7:** Performance comparison of factory and own calibrations for direct regression approach of sensors from ECH_2_O family: *R*^2^ values, average AD (vol %) and RMSE values (vol %).

			Factory Linear Calibration			Own Linear Calibration		
Sensor Type	Material	Packing	*R*^2^	Average AD (vol %)	RMSE (vol %)	*R*^2^	Average AD (vol %)	RMSE (vol %)
ECH_2_O TE	Soil	Moderate	0.962	2.25	2.88	0.962	1.98	2.31
ECH_2_O EC-5	Soil	Moderate	0.977	2.97	3.07	0.977	1.54	1.80
ECH_2_O EC-10	Soil	Moderate	0.968	4.83	5.81	0.968	1.91	2.12
ECH_2_O EC-20	Soil	Moderate	0.972	4.84	5.83	0.972	1.81	1.97
ECH_2_O TE	Sand	Loose	0.963	1.34	3.68	0.992	0.49	0.62
ECH_2_O EC-5	Sand	Loose	0.982	3.40	5.85	0.982	0.77	0.93
ECH_2_O EC-10	Sand	Loose	0.926	13.06	16.43	0.928	1.45	1.86
ECH_2_O EC-20	Sand	Loose	0.811	11.90	15.08	0.810	2.17	3.02
ECH_2_O TE	Sand	Moderate	0.983	1.27	2.80	0.983	0.95	1.11
ECH_2_O EC-5	Sand	Moderate	0.986	1.56	3.28	0.986	0.85	0.98
ECH_2_O EC-10	Sand	Moderate	0.917	11.81	15.05	0.917	2.07	2.43
ECH_2_O EC-20	Sand	Moderate	0.930	11.26	13.93	0.930	1.96	2.23
ECH_2_O TE	Sand	Compact	0.976	1.35	2.16	0.978	1.19	1.44
ECH_2_O EC-5	Sand	Compact	0.981	1.29	2.61	0.981	1.09	1.32
ECH_2_O EC-10	Sand	Compact	0.977	7.18	10.02	0.963	0.38	0.49
ECH_2_O EC-20	Sand	Compact	0.993	7.87	10.02	0.991	0.75	0.90

**Table 8:** Performance comparison of factory and our own linear calibrations for all five tested sensors in saline conditions: average AD (vol %) and RMSE values (vol %).

				Factory Linear Calibration		Own Linear Calibration	
Sensor Type	Material	Packing	Salinity ^1^	Average AD (vol %)	RMSE (vol %)	Average AD (vol %)	RMSE (vol %)
ECH_2_O TE	Sand	Compact	1st round	0.64	0.72	0.42	0.62
ECH_2_O EC-5	Sand	Compact	1st round	2.81	2.90	0.92	0.95
ECH_2_O EC-10	Sand	Compact	1st round	4.03	4.42	10.73	11.51
ECH_2_O EC-20	Sand	Compact	1st round	4.09	4.49	12.95	13.66
ThetaProbe	Sand	Compact	1st round	1.32	1.63	2.00	2.03
ECH_2_O TE	Sand	Compact	2nd round	1.82	1.93	0.93	1.07
ECH_2_O EC-5	Sand	Compact	2nd round	1.75	2.37	1.41	1.43
ECH_2_O EC-10	Sand	Compact	2nd round	11.11	12.23	20.55	21.43
ECH_2_O EC-20	Sand	Compact	2nd round	10.36	11.22	21.25	21.79
ThetaProbe	Sand	Compact	2nd round	3.79	4.31	4.65	4.79
ECH_2_O TE	Sand	Compact	3rd round	4.63	4.71	3.49	3.52
ECH_2_O EC-5	Sand	Compact	3rd round	1.80	1.97	2.53	2.91
ECH_2_O EC-10	Sand	Compact	3rd round	16.74	17.12	26.62	26.87
ECH_2_O EC-20	Sand	Compact	3rd round	16.06	16.45	27.39	27.61
ThetaProbe	Sand	Compact	3rd round	7.58	7.64	7.70	7.71

^1^ Increasing salinity level due to repeated saline water application on the sand material; 1st round = the initially dry sand was wetted by saline water, then the water was evaporated (salt remained) and new wetting with saline water started = 2nd round, etc.
